# Concepts, abstractness and inner speech

**DOI:** 10.1098/rstb.2021.0371

**Published:** 2023-02-13

**Authors:** Anna M. Borghi, Charles Fernyhough

**Affiliations:** ^1^ Department of Dynamic and Clinical Psychology, and Health Studies, Sapienza University of Rome and Institute of Cognitive Sciences and Technologies, Italian National Research Council, Rome 00185, Italy; ^2^ Department of Psychology, Durham University, South Road, Durham DH1 3LE, UK

**Keywords:** ‌, abstract concepts, abstract words, inner speech, metacognition, dialogic inner speech, conceptual acquisition

## Abstract

We explore the role of inner speech (covert self-directed talk) during the acquisition and use of concepts differing in abstractness. Following Vygotsky, inner speech results from the internalization of linguistically mediated interactions that regulate cognition and behaviour. When we acquire and process abstract concepts, uncertainties about word meaning might lead us to search actively for their meaning. Inner speech might play a role in this searching process and be differentially involved in concept learning compared with use of known concepts. Importantly, inner speech comes in different varieties—e.g. it can be expanded or condensed (with the latter involving syntactic and semantic forms of abbreviation). Do we use inner speech differently with concepts varying in abstractness? Which kinds of inner speech do we preferentially use with different kinds of abstract concepts (e.g. emotions versus numbers)? What other features of inner speech, such as dialogicality, might facilitate our use of concepts varying in abstractness (by allowing us to monitor the limits of our knowledge in simulated social exchanges, through a process we term *inner social metacognition*)? In tackling these questions, we address the possibility that different varieties of inner speech are flexibly used during the acquisition of concepts and their everyday use.

This article is part of the theme issue ‘Concepts in interaction: social engagement and inner experiences’.

## 1. Introduction

We use concepts to store knowledge, make inferences and orient ourselves in the world. Concepts can differ in their abstractness level (e.g. ‘truth’ versus ‘table’). In this article, we explore the role of inner speech (covert self-directed talk) during the acquisition and use of concepts differing in abstractness. We start by examining the distinction between concrete and abstract concepts and illustrating some evidence on electromyographical activation of articulatory muscles during abstract concept processing, possibly suggesting the use of inner speech. We then describe some recent advances in inner speech research, particularly around the use of different kinds of inner speech. We then outline our proposal, detailing how and when inner speech might be used during the acquisition and processing of different kinds of abstract concepts. We emphasize that our aim in this paper is not (yet) to build a novel theoretical model but to start exploring the connections between two research areas that so far have been entirely distinct, outlining some mechanisms that might be at play. Finally, we point to new research directions in this fascinating and rather unexplored area.

### (a) Abstract concepts

Historically there has been a progression away from viewing the distinction between abstract and concrete concepts as a dichotomy toward a view of them as representing points on a continuum. We prefer to think of them as occupying a multidimensional space in which more concrete and more abstract concepts vary along different dimensions. Abstract concepts are generally acquired later in development, in a process scaffolded by language rather than by communicative gestures such as pointing (linguistic 'Modality of Acquisition, MoA) [1]. Typically, abstract concepts are relational constructs [2]. Compared with concrete concepts, they are more complex, involving more elements and their relations [3], and are also morphologically different [4]. Furthermore, they are generally less iconic [5], more detached from sensory modalities but more linked to interoception [6,7], and more emotionally charged [8,9]. These dimensions might be differently relevant depending on the kind of abstract concept involved: for example, for emotional concepts, the role of interoception might be prominent, but it may not be for numerical concepts [10]. Notably, for the acquisition and use of all kinds of abstract concepts, linguistic and social interaction might be particularly crucial because of the heterogeneity and semantic dissimilarity of the concepts involved: perceptual input might be insufficient to form them, while linguistic labels and explanations might be profoundly beneficial in understanding them. On this view, linguistic labels could work as a sort of glue [11] that helps to keep together perceptually dissimilar category members. The important role in processing abstract concepts played by linguistic, social and emotional dimensions is one of the more significant developments attributed to so-called multiple representation views (e.g. [12–17]; for a review, [18]).

### (b) Mouth effector involvement

Positing that linguistic experience is crucial for abstract concept representation, while sensorimotor experience is of comparable importance for concrete concepts, has led authors to investigate the different effector systems that different kinds of concepts involve. Abstract and concrete word processing might involve different effector systems: evidence shows that the hand effector is more involved during the processing of concrete concepts and, within abstract concepts, of numerical ones, while the mouth effector has a comparable role during the processing of more abstract concepts, particularly mental state ones [19,20]; for an overview see [21]. In recent work, Reggin *et al*. [22] investigated age of acquisition norms and found an interaction between word concreteness and the involvement of the mouth effector: abstract words rated as having lower mouth action strength, i.e. being ‘less experienced with mouth actions' (see Lancaster sensorimotor norms [23]), are acquired later than concrete words with lower mouth action strength. Specifically, in recent studies with different paradigms (e.g. definition matching and word recognition tasks), one of the present authors found evidence of facilitation when adult participants responded to abstract concepts using the mouth (e.g. responding ‘yes’ by using a microphone, or squeezing a device between their teeth) (review in [24]). Similarly, facilitation occurred when participants responded to concrete concepts using the hand (e.g. pressing a button on the keyboard, or squeezing a device with the fingers).

Despite this evidence, it is still possible that the activation of the mouth could simply be a by-process and not necessary for conceptual comprehension. To this aim, we designed some studies using interference paradigms. First, Barca *et al*. [25,26] investigated in children whether the long-term use of pacifiers might selectively interfere with the acquisition of abstract concepts and have a long-lasting effect in different processing tasks. Results from a definition task in 6-year-olds and a categorization task in 8-year-olds suggested that occupying the mouth during word acquisition might have a specific effect on subsequent abstract concept representation and processing, leading to a more blurred distinction of the conceptual relations evoked by concrete and abstract concepts and yielding longer response times with abstract concepts. Other studies adopted interference paradigms in online tasks performed by adults, asking participants to pronounce a syllable (articulatory suppression) while performing a different task. Villani *et al*. [7] found no effect of articulatory suppression on difficulty ratings: articulatory suppression increased the perceived difficulty of both concrete and abstract words. In another study by one of the present authors, participants had to continuously pronounce a syllable (articulatory suppression) and squeeze a ball while categorizing words as concrete or abstract [27]. The results, also confirmed by a drift-diffusion model, showed that articulatory suppression, but not ball squeezing, slowed down response times more with abstract than with concrete concepts. One could object that a demanding task, articulatory suppression, would interfere more with the more difficult task, abstract concept processing, than with the easier task of squeezing a ball. The authors argue that this is likely not the case because the words were balanced for age of acquisition, generally correlated to word difficulty. However, further research is needed to understand better whether and to what extent articulatory suppression impairs abstract concept processing. Two issues should be discussed.

First, one might wonder why inner speech, as selectively modulated by a continuous task like articulatory suppression, should impact upon a task occurring quickly in time, such as a word categorization one. A possible response lies in arguing that inner speech might be condensed; for example, we might drop some words and abbreviate others [28], and we do not require time for breathing (see evidence by Korba [29] on inner speech during problem-solving) (for an overview, see [30]).

Second, one might object that it is obvious that articulatory suppression, a rather superficial linguistic task, does not interfere with inner speech elicited during abstract concept processing. It is, instead, possible that deeper forms of verbal interference play a major role and that articulatory suppression impairs only superficial articulation. The issue at stake is related to current debates on inner speech: it is unclear whether inner speech is necessarily articulated [31]. On the one hand, according to an embodied perspective, both inner and overt speech activate a motor simulation, even if during inner speech such a simulation would not lead to the production of sounds. Such a simulation would, however, involve motor planning and articulation, even if no execution. The so-called ‘abstraction view’ (e.g. [32]) contends that such a simulation concerns planning but no execution, and, in some versions of this view, inner speech would be specified neither at the articulatory nor at the acoustic level.

According to a different view (e.g. [33,34]), inner speech would, in certain contexts, be less rich and different from outer speech since, depending on the situation, a more extended or condensed form would be activated. If inner speech is condensed, then full articulation would not be necessary; multiple coexisting levels of abstraction would thus characterize inner speech. Oppenheim & Dell [35] propose that speakers can monitor the degree of articulation of their speech—similarly to what we do with outer speech, passing from shouts to whispers, we can flexibly modulate our inner speech depending on the context and situation. Hence, articulation would not be necessary for inner speech, but interference with it by the articulatory suppression task would determine a change in our use of inner speech.

Note that, differently from other authors, we do not think that the fact that inner speech in some circumstances is not articulated necessarily implies that it is ‘abstract’ or ‘amodal’. Our focus in this paper is not the format of inner speech *per se* (which is likely multifarious, with considerable individual differences), but the extent to which inner speech can contribute to creating, processing, and using concepts, and particularly more difficult and abstract concepts. Conceptual meaning can be grounded in perception and action systems, even if these concepts are conveyed through a non-articulated form of inner speech.

In sum: evidence shows that the mouth motor system is more involved during abstract than concrete concept acquisition and processing. With abstract concepts, mouth responses are facilitated, even if it is not always the case—for example, we found no mouth response advantage in the lexical decision tasks on single words, probably because the processing level is too shallow. Furthermore, studies showed that extensive pacifier use might interfere with the acquisition of abstract concepts and that articulatory suppression might delay their processing. The evidence that the mouth motor system is more activated with abstract than concrete concepts does not allow concluding that articulation is necessary for abstract concept acquisition and processing. However, it testifies that linguistic experience is likely important for abstract concepts, with the mouth motor system activation as a proxy for language involvement. The stronger activation of the mouth motor system suggests that language, and possibly inner language, is used to access the conceptual meaning. As we will detail in §2, we might use different kinds of inner speech endowed with different functions during conceptual acquisition and word processing. Notably, the relevance of the mouth effector holds only for spoken languages; it is possible that, in the case of sign languages, the activation of the mouth motor system is substituted by that of the hand motor system. A similar argument can be made for inner speech: if mouth/lip movements are detected, it can be a strong clue that inner speech is present. But not detecting such movements should not be taken to imply that there is no inner speech.

Given the apparent importance of language in conceptual processing, it becomes critical to determine which kind of language and which functions of language are more crucial for the acquisition, representation and use of concepts, particularly abstract ones.

We detail below our proposal for involvement of inner speech, which might differ during the acquisition and the use of concepts, particularly abstract ones. We mainly outline some possible hypotheses that should be tested through further research.

### (c) Varieties of inner speech

Following Vygotsky [28], we view inner speech as resulting from the internalization of linguistically mediated interactions that are used to regulate cognition and behaviour. Although there remain gaps in knowledge, this view of the development of inner speech has been supported by evidence from children's private speech (or out-loud self-talk; e.g. [36]) as well as by studies with typically developing children and adult participants and neurodiverse groups [34]. Inner speech is now recognized to take different forms, in line with its origins in social interaction [37], with features such as dialogicality and condensation reliably appearing as factors in self-report studies [34,36–38]. With particular relevance to this article, two factors in the latest version of the VISQ-R inner speech self-report instrument may be particularly significant for learning abstract concepts, as we explain below: namely the factors of *evaluative/critical* inner speech and *positive/regulatory* inner speech [34,36–38].

## 2. Inner speech and abstract concept acquisition and processing

### (a) Inner speech during conceptual acquisition

To learn abstract concepts, the input of others conveyed through outer speech is fundamental. Studies on the acquisition of novel abstract concepts have highlighted how explanations of concepts by others are particularly important [39]. Unlike concrete concepts, abstract ones are low-dimensional, i.e. their members are highly heterogeneous. This clarifies why it may be very difficult to learn them based on perceptual input; the support of others, which may consist of linguistic explanations, is, therefore, pivotal for acquiring them. Consistently, they are acquired later than concrete concepts and predominantly through the linguistic modality (Modality of Acquisition, MoA, [1]).

During this acquisition phase, inner speech might perform different functions. While that of rehearsing and inwardly repeating the word meaning might not be specific to abstract words but extend to concrete ones [40], it is possible that we might need to rehearse more newly acquired abstract words in order to codify and consolidate them in memory because they are more crucial for keeping together the category members. This might occur for various reasons, including the fact that abstract words are less iconic because less richly perceptually featured.

Consider research intending working memory as a continuous resource rather than as being constrained by a fixed number of slots [41]. Studies show the advantages of visually salient stimuli and indicate that the noise level increases with the increase of the number of stimuli, provoking a decline in working memory precision. Consistent with this, the noise level might be higher for concepts collecting heterogeneous exemplars and whose members are less visually salient, like abstract concepts. Because the to-be-acquired label refers to multiple exemplars, we might need to repeat it more. Hence, rehearsal of the label associated with the category members through inner speech might be particularly crucial when encoding novel abstract words and might support memory consolidation. Consistent evidence with working memory of written stimuli shows that non-optimal language comprehension does not reduce the sensitivity to the phonological aspects of words but rather leads to a limited recall of abstract concepts [42].

One might object that such a process would occur for any concept that is challenging to grasp, such as ‘oak tree,’ and is not specific to abstract concepts. As anticipated in the Introduction, we do not believe there is a dichotomy between concrete and abstract concepts, but rather that different kinds of concrete and abstract concepts are differently arranged in the multidimensional space, with some dimensions weighing more than others. At the same time, we need adequate tools for complicated matters. Hence, we posit that inner speech will be more engaged the greater the difficulty of the word and the greater the ease of access to its referents. Whereas ‘oak tree’ is certainly more difficult to learn than ‘dog,’ its difficulty lies more in detecting the features that distinguish it from other trees than in grouping together heterogeneous members, as is the case with more abstract concepts. Concrete concepts typically have single objects or entities as referents; hence it is easier to form cohesive concrete categories. Therefore, we hypothesize that complex concrete concepts like ‘oak tree’ will engage inner speech more than easy ones but less than abstract concepts and that some kinds of abstract concepts, like philosophical–spiritual ones, engage inner speech more than others. Consistent evidence comes from neuroscientific literature, showing stronger activation of areas typically associated with inner speech (e.g. [43]) and the more marked activation of the left inferior frontal gyrus during abstract concepts processing [44,45].

Another interesting point for future research is determining whether we resort to different kinds of inner speech in the conceptual acquisition phase compared with later ones, i.e. using the concept acquired. It is possible that, during conceptual acquisition, we need to use an expanded form of inner speech to explain to ourselves in detail the meaning of words, while, during word use, a more abstract, condensed form might suffice. Hence, expanded and condensed forms are not necessarily mutually incompatible, but they might be related to two different processes. In learning and acquisition, an expanded form might be used, while in abstract concept use, a condensed form might be more effective.

### (b) Inner speech during conceptual use

The greater difficulty of abstract words, also evidenced by the later age of acquisition in children, results in the well-known concreteness effect, i.e. the longer reaction time in processing abstract compared with concrete words [46] and the greater difficulty in recalling abstract compared with concrete terms. The causes of the concreteness effect have been ascribed variously: to the fact that abstract concepts are linguistically coded, in the framework of the dual code theory (DCT) [46]; to the fact that abstract concepts evoke many contexts but are less tightly linked to a specific context, in the framework of the context availability theory (CAT) [47]; or to the fact that abstract concepts are characterized by a lower degree of perceptual strength [48].

Whatever the explanation of the concreteness effect, the use of abstract words, which express abstract concepts, typically involves uncertainty. This is particularly evident when, while reading or engaging in a conversation, we encounter an abstract word. In a study in which participants responded to a sentence involving different kinds of concrete and abstract concepts, we found clear signals of this uncertainty. When participants had to respond to sentences involving abstract concepts, and particularly more abstract ones, i.e. philosophical–spiritual ones, they used expressions of uncertainty more often and asked more How and Why questions. By contrast, with concrete concepts, Where and What questions were more frequent. For example, participants used expressions like ‘What do you mean? Why? Explain to me better’ [49]. The use of such expressions testifies that participants use metacognition in assessing their knowledge. A monitoring process is likely in play, in which participants control a cognitive activity, in this case their mastery of the conceptual content [50]. We intend this inner monitoring as a form of second-order thought [51], a process by which we reflect on our thinking process and the content of our knowledge and evaluate it. Whether this form of metacognition is explicit has to be determined; certainly, it has an explicit outcome since it is expressed in words. Further rating evidence corroborates that a metacognitive process is in play when we process abstract words and that it might be explicit: compared with concrete words, participants feel less confident in the word meaning with more abstract words, feel less confident that even the experts know the word meaning [52], and feel they need the help of others more to learn the word meaning [10]. This feeling of uncertainty is the product of an evaluation of our own knowledge and is therefore metacognitive. Basically, we explicitly evaluate our (abstract) concepts as full of gaps. Notably, one could object that this feeling of uncertainty following a monitoring process might occur with many different concepts, particularly difficult ones. As previously explained, we believe that the distinction between concrete and abstract concepts is nuanced. Hence, we think that this uncertainty might characterize various concepts, but to a larger extent those that refer to heterogeneous members and the meaning of which is more indeterminate and negotiable, like abstract concepts [51,53], and in particular for the more abstract among these concepts.

We propose that inner speech plays a crucial function in coping with the uncertainty that these concepts elicit. Importantly, we propose that different functions of inner speech might be exploited in various contexts, with different kinds of inner speech activated (figure 1). This proposal should be tested and verified in light of experimental evidence. To test the involvement of articulated inner speech, articulatory suppression and other verbal interference tasks could be employed, including surface electromyography (EMG) (but see [52,54]) and transcranial magnetic stimulation (TMS). To test which kind of inner speech is activated, questionnaires focused on varieties of inner speech could be particularly useful (e.g. [38]), combined with analysis of whether different abstract concept acquisition and processing tasks engage brain areas related to different kinds of inner speech [55].

**Figure 1.**
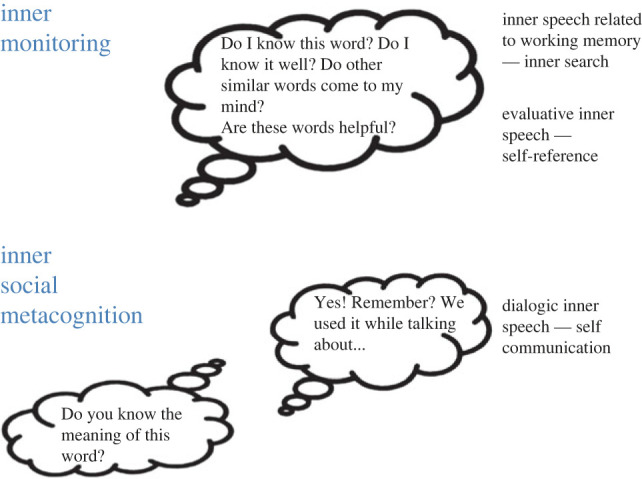
The processes of inner monitoring and inner social metacognition, and the kinds of inner speech that might accompany them.

First, different varieties of inner speech might contribute to the inner monitoring process in various ways. During this inner monitoring phase, a form of inner speech related to verbal working memory might be used [56]. When we hear or read a word the meaning of which we are not certain, we might rehearse possible other words to trace its meaning [57]. The presence of this process of word search is compatible with the activation of the left inferior frontal gyrus, often associated with phonological working memory and other high-level motor control functions, during abstract concept processing [49,50]. Note that we do not think this process is merely phonological, but that words retrieved through rehearsal can carry meaning and help people retrieve or reconstruct the conceptual meaning or even re-explain it to themselves.

One possibility is that the evaluative function of inner speech could play a role [37] in contributing to judgements about the adequacy and richness of the content of our concepts and assessing the effectiveness of the word search results. Researchers have highlighted the contributions of inner speech to self-reflection, self-regulation and self-knowledge, even in situations where this self-knowledge might be inaccurate [58].

Suppose, however, that the word search process is unsuccessful. How do we become able to understand and use abstract concepts, capturing their distinctive aspects—e.g. fine-grained differences such as those between *shame* and *modesty*? One way to do this is through deference [50], i.e. referring to authoritative and competent others and tracing the meaning through their help [59]. This is the process we have elsewhere termed social metacognition [60]. The use of inner speech may play a role in monitoring concept meanings, but in cases where this monitoring leads us to detect missing aspects, we might need to refer to other people, as typically happens in interpersonal contexts in which testimony is sought and evaluated [61,62]. Importantly, these other people might not be actual, present others but instead may figure in a form of dialogic inner speech.

Inner speech can help us to prepare to refer to others, to ask them to fill our knowledge gaps. More intriguingly, inner speech can also serve to simulate the presence of others. While social metacognition would involve the tendency to revert to actual, physically present others [63], what we here term *inner social metacognition* would involve the tendency to dialogue with ourselves to find possible answers. As the research summarized above suggests, inner speech can take a dialogic form, allowing us to have back-and-forth conversations with non-present or even imaginary others [38,55,64]. This process is intriguing for at least two reasons. First, it makes sense of the intuition that self-communication is genuine communication, at least in the sense of communicating knowledge to a recipient that that individual does not apparently possess [62]. Basically, we can furnish answers to ourselves that we do not believe ourselves to have. Second, it might highlight the power of language in showing that the solution we cannot directly reach can be achieved through argumentation.

## 3. Varieties of inner speech, varieties of abstract concepts

So far, we have spoken in terms of a general distinction between abstract and concrete concepts. However, the most recent trends in the area show that abstract concepts incorporate multiple dimensions and that, depending on the kind of concepts, some dimensions are more relevant to their representation than others. For example, Villani *et al*. [10] collected ratings of 15 dimensions (e.g. imageability, contextual availability, perceptual strength, inner grounding, social metacognition, etc.) and found through principal component analysis and cluster analysis that abstract concepts can be divided into four sub-kinds. These different kinds of abstract concepts range from the more concrete physical space–time and quantity concepts, PSTQ (e.g. acceleration, effort), for which the sensorimotor aspects are more crucial, to the more abstract philosophical and spiritual concepts (e.g. fate, morality), to the concepts of self and sociality (e.g. kindness), grounded both in the sensorimotor and inner dimension (interoception, metacognition), and finally to emotional and inner states concepts (e.g. love), for which inner grounding plays a major role. Similarly, Harpaintner *et al*. [15] determined (on the basis of a feature-listing task) that some abstract concepts are characterized by verbal associations, some by a high proportion of internal/emotional features, and others by a large proportion of sensorimotor features. In a recent review, Conca *et al*. [66] examined 40 studies on kinds of abstract concepts published until 2020. They found that the four concepts more often distinguished are emotional concepts, mental state concepts, social concepts and numerical concepts; for each, different dimensions assume relevance. Hence, different varieties of abstract concepts exist, and they can be represented as different points in a multidimensional space [67,68].

Provided that abstract concepts are not a unitary whole, it would be important to determine whether they involve inner speech to a different extent and whether different varieties of inner speech accompany or perhaps even facilitate them. Overall, we hypothesize that inner speech might be particularly crucial when sensorimotor input is lacking; therefore, we predict its use will be more extensive with more abstract concepts, like philosophical–spiritual ones, than other abstract concepts. It is also possible that a more condensed form of inner speech is used during the processing of numerical concepts, which participants generally evaluate as less abstract. By contrast, we might use more expanded forms when we need to re-explain to ourselves conceptual meaning, as in the case of more abstract concepts like philosophical–spiritual ones. As to emotional concepts, they may activate different kinds of inner speech depending on their content: for example, we might use evaluative/critical or positive/regulatory inner speech more extensively with emotional concepts like fear or shame, where inner speech can contribute to controlling our behaviour, while dialogic inner speech might be used more with concepts like love and affection, where we re-enact previous dialogic experiences or simulate novel ones.

Although at this stage it remains speculative, it is also interesting to consider individual differences in inner speech and abstract concept use. Individual differences in inner speech use have been shown to relate to self-awareness and self-evaluation [69,70]. The concepts that mediate self-knowledge are likely to be predominantly abstract ones (concerning emotions, relationships, personality factors, etc.). This raises the possibility that the observed connections between inner speech and self-knowledge might be mediated by particular subtypes of inner speech relevant for operating with abstract concepts. Future research might fruitfully address these empirical questions.

## 4. Conclusion

Abstract concepts come in different varieties, and different varieties of inner speech exist. Starting from the assumption that language deeply impacts cognition, we have sought in this article to explore how different kinds of inner speech can be employed during the acquisition and use of different kinds of abstract concepts. Curiously, so far the intersection between concepts, particularly abstract ones, and inner speech has been a rather unexplored area. Addressing it might open new, fascinating research avenues.

## Data Availability

This article has no additional data.
